# Transillumination method in total knee arthroplasty: new approach with old tools

**DOI:** 10.1007/s00264-023-05977-6

**Published:** 2023-09-13

**Authors:** Murodulla Karimov, Sarvar Madrakhimov

**Affiliations:** Department of Traumatology and Orthopedics, Tashkent Medical Academy, Tashkent, Uzbekistan

**Keywords:** Patella, Lateral retinaculum release, Blood supply, Total knee arthroplasty

## Abstract

**Purpose:**

In this study, we evaluated the possibility of precise intraoperative localization of the lateral genicular arteries by an orthopaedic surgeon using the transillumination method.

**Methods:**

Twelve patients underwent cemented TKA with patella-friendly Zimmer Biomet NexGen Legacy Posterior Stabilized prostheses (without patellar resurfacing), seven right knees and five left knees. The mean age of patients in the study group was 66.636 ± 7.003 years. The minimal follow-up period was 13 months (mean—16.363 ± 2.5 months). Functional outcomes were assessed using Knee Society and a specific patellar questionnaire—Kujala Score. Intraoperative detection of insufficient patellar stability and/or patellar maltracking was based on the no-thumb technique. In pre- and postoperative period X-ray investigation, standard standing X-ray and Merchant view were used to evaluate implant position and patellofemoral congruency.

**Results:**

In this study, ten out of twelve knee joints (83.3%) had at least one artery visible by the proposed method in the lateral parapatellar area. Five out of ten knee joints had more than one artery that could be visualized and identified as an arterial vessel. Postoperative Knee Society Score showed significant improvement from a mean 51.181 ± 3.868 to a mean 88.727 ± 3.663. Mean hospital length of stay is 8.545 ± 1.863 days. X-ray assessment using standard anteroposterior, lateral, and Merchant skyline views showed appropriate implant positioning and patellofemoral congruency. The mean Kujala Score in the postoperative period (3 and 6 months) was 67.3 ± 6.75 and 75.6 ± 6.42, respectively.

**Conclusions:**

Using the proposed transillumination method can help preserve the lateral blood supply to the patella and to avoid devascularized patella-related complications.

**Trial registration:**

Retrospectively registered on 5 of May 2023, Registration number – 3/3-1757.

## Introduction

Total knee arthroplasty (TKA) is one of the most common surgical procedures performed in orthopaedics over the past decades [1, 2]. Usually, TKA is recommended as the only surgical management of end-stage degenerative osteoarthritis (OA), in which all three compartments of the knee are affected. During this surgery, the medial parapatellar approach is the most popular [3] where medial genicular arteries (aa. inferior et superior medialis genicularis) are inevitably sacrificed. These vessels are of great importance for the blood supply of the patella due to their larger diameter and the number of branches participating in the formation of the peripatellar anastomotic ring [4].

Patella-related complications are quite common in TKA [5, 6]. Despite improvements in the design of the implants, these complications are responsible for 10% of all TKA revisions [7, 8]. The most common complications are stress fractures of the patella and loosening of the prosthesis components (in TKA with patella resurfacing) [9], which can be manifestation of hypo- and devascularization of the patella [10].

Another critical complication is patellofemoral instability, which varies from 1 to 20% of all patellar complications [5, 11, 12]. Intraoperative detection of this condition results in the lateral release of the patellar retinaculum in up to 45% of cases [13, 14]. Lateral release significantly increases the chance of the lateral genicular arteries injury and consequently may lead to iatrogenic devascularization of the patella [15, 16].

The aim of this pilot study was to evaluate the possibility of precise intraoperative localization of the lateral genicular arteries (aa. superior et inferior genicularis lateralis) by an orthopaedic surgeon using the transillumination method and subsequently to leave these vessels intact during the lateral release of the retinaculum. The next goal was to assess the probability of matching the projection of lateral vessels with routine incisions, which potentially could damage the abovementioned vessels.

## Materials and methods

From January 2022 to May 2022, 63 patients with end-stage primary osteoarthritis of the knee joint [17] underwent primary TKA in a single centre, 12 of them (4 men, 8 women) had indications for intraoperative release of lateral retinaculum due to intraoperatively detected patellofemoral instability (TKA was performed without the usage of tourniquet). None of the patients had a history of patellofemoral instability nor radiographic evidence of instability factors before TKA. All 12 patients underwent cemented TKA with patella-friendly Zimmer Biomet NexGen Legacy Posterior Stabilized prostheses (without patellar resurfacing), seven right knees and five left knees. The mean age of patients in the study group was 66.636 ± 7.003 years. The minimal follow-up period was 13 months (mean—16.363 ± 2.5 months). Functional outcomes were assessed using Knee Society and a specific patellar questionnaire—Kujala Score. The procedure of implantation of femoral and tibial components included standard protocol related to patellar tracking. For femoral rotation, we set 3° of external rotation. In terms of tibial component implantation, the anatomical landmark utilized was the junction of the medial and central thirds of the tibial tuberosity. As a secondary reference point, the tibial external axis extending to the second metatarsal bone was also utilized. Intraoperative detection of insufficient patellar stability and/or patellar maltracking was based on the “no-thumb technique” followed by re-evaluation with “towel clip technique” [18]. In pre- and postoperative period we utilized X-ray investigation, including standard standing X-ray and Merchant view for implant positioning and patellofemoral congruency assessment.

The lateral release was performed under the transillumination control which determined the exact localization of lateral genicular arteries. The time required for the procedure took on average 4.2 ± 1.3 min. In order to prevent an increase of infection-related complications, we decided not to go beyond the time frame of five min while searching lateral genicular arteries, dictated by several studies that showed that periprosthetic risk infection risk gradually increases by prolongation of operation time every six to 15 min [19, 20]. The result was considered negative when the arteries were not found within five min.

Doppler ultrasound sonography was performed by an experienced doctor (US Machine) in the postoperative period (1-day postop) for blood supply control. The task of the ultrasound doctor was to determine the blood supply to the lateral parapatellar area, which can be presented by two arteries. A search was made for all possible variations [21], when one artery was found, the search continued until the absence of other arteries was confirmed.

### Transillumination method of visualization of parapatellar vessels

**Fig. 1:**
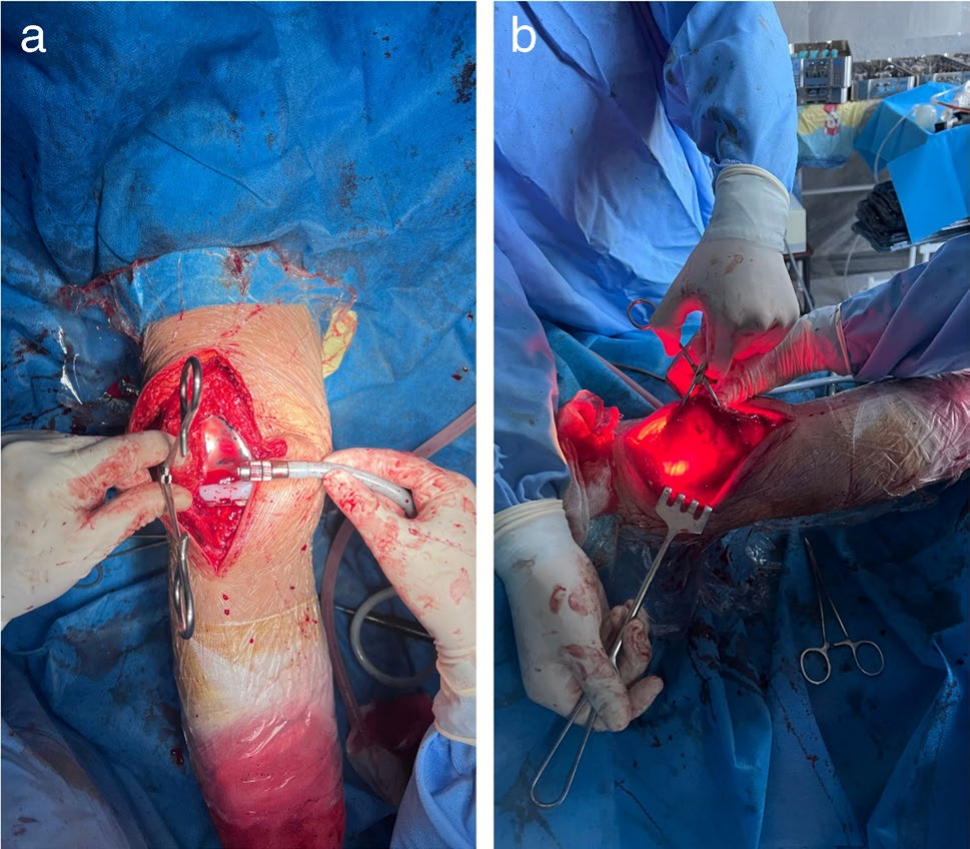
Frontal (**a**) and lateral (**b**) views of the right lower extremity after total knee arthroplasty during preparation for transillumination of lateral parapatellar area

**Fig. 2:**
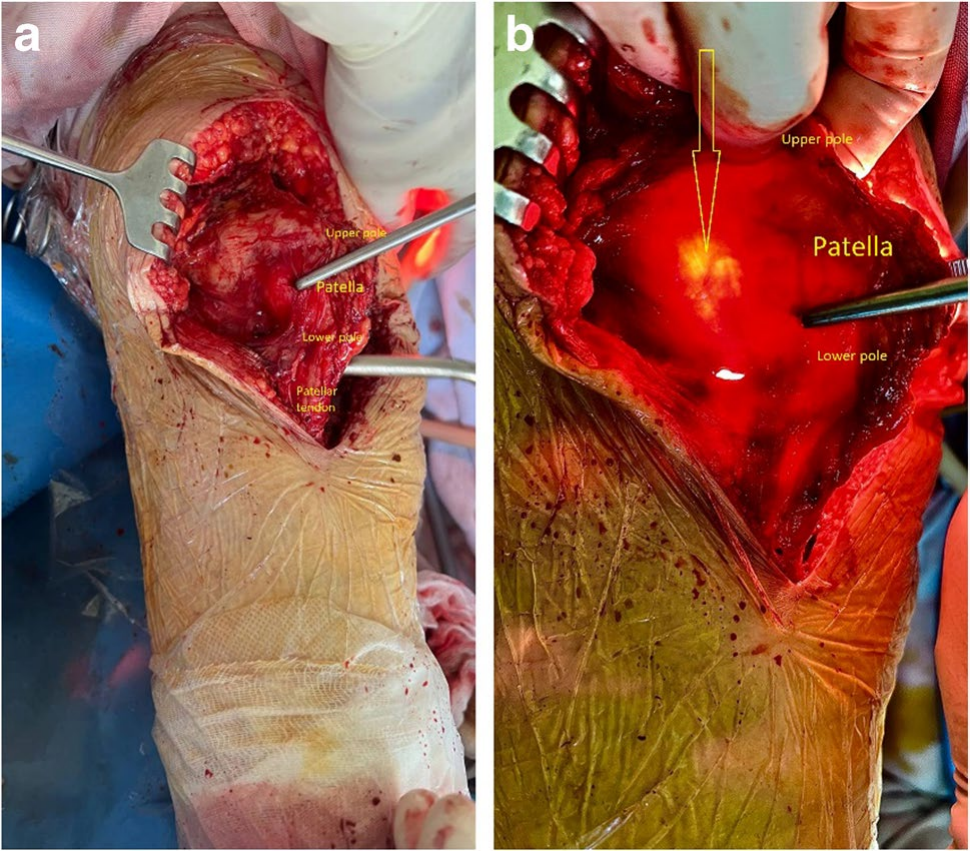
Transillumination method: light off (**a**) and light on (**b**) modes. The yellow arrow indicates the superior lateral genicular artery

In our study, we utilized the light of Storz Xenon 300 Light Source for Endoscopy, the tip of which is tightly inserted into the intra-articular side of the lateral retinaculum, while lifting and everting the patella outwards in 90° with towel clips in the medial edge (the limb is in 180° of extension) (Figs. 1 and 2), while the assistant makes fan-like movements from top to bottom and medial to lateral. The arteries are visualized as dark red and identified in the upper-lateral and lower-lateral directions lateral retinaculum, after which the direction of the arteries is marked with a marker. Next, the lateral retinaculum is released distally from the marked edges to the blood vessels, which remain intact.

## Results

**Fig. 3:**
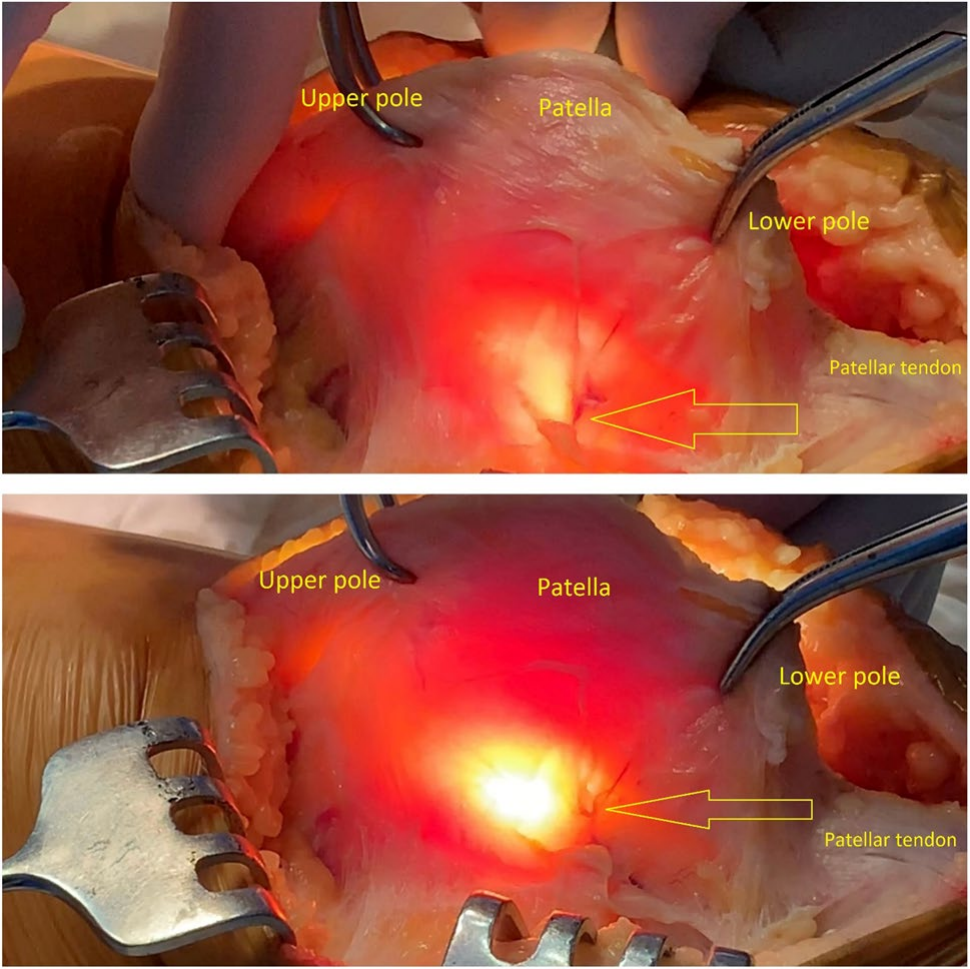
Clearly visualized inferior lateral genicular artery (yellow arrow)

In this study, ten out of twelve knee joints (83.3%) had at least one artery visible by the proposed method in the lateral parapatellar area (Fig. 2). Five out of ten knee joints had more than one artery that could be visualized and identified as an arterial vessel. In those five cases, we were able to identify two arteries (aa. lateralis genicularis superior et inferior) (Fig. 3). With the lateral release of the lateral retinaculum, out of the 10 cases identified, all cases were performed with high caution and preservation of the lateral patellar blood supply.

In two cases (16.7%) out of twelve, the lateral release was performed without finding and identifying lateral genicular arteries. In those cases, the release was carried out only in the potentially safe area of the lateral retinaculum (15 mm from the edge of the patella from the top to the bottom) [4].

In postoperative Doppler ultrasound sonography, we found at least one artery, namely superior lateral genicular artery (SLGA) in all 12 patients; eight out of 12 patients had also inferior lateral genicular artery (ILGA). The mean diameter of SLGA was 1.88 ± 0.244 mm; ILGA mean diameter was 1.31 ± 0.18mm. The Doppler ultrasound sonography in the one day postoperative period in patients with identified vessels by the proposed method in all cases confirmed preserved blood flow. Two cases where the transillumination method was not helpful in finding arteries lateral patellar blood supply were presented by SLGA.

Eight patients (5 women, 3 men) out of 12 had mild valgus deformity; the rest of the patients had mild varus deformity. The mean preoperative Knee Society Score was 51.181 ± 3.868. Postoperative Knee Society Score showed significant improve from a mean 51.181 ± 3.868 to a mean 88.727 ± 3.663. Mean hospital length of stay is 8.545 ± 1.863 days. X-ray assessment using standard anteroposterior, lateral, and Merchant skyline views showed appropriate implant positioning and patellofemoral congruency. The mean Kujala Score in the postoperative period (3 and 6 months) was 67.3 ± 6.75 and 75.6 ± 6.42, respectively.

## Discussion

Blood supply to the patella is carried out due to the anastomotic ring, the so-called rete patellaris, formed from five to eight arteries. It is worth noting that the main role in blood supply is played by lateral and medial genicular arteries. Damage to these vessels correlates with severe postoperative patellar complications after even technically ideal TKA. Several authors classified patellar devascularization as a surgical technique-related risk factor for periprosthetic patellar fractures following lateral retinaculum release during TKA [22, 23].

Intraoperative patellofemoral instability must be eliminated to avoid complications such as anterior knee pain syndrome, limitation of range of motion, elongation of rehabilitation time [24], subluxation, and luxation of the patella. Instability, which occurs even with technically correct alignment of the limb axis and implants of the femoral and tibial components, forces the surgeon to perform the procedure of lateral release of the patellar retinaculum.

The existing recommendations for the implementation of lateral release in TKA [14, 25–27], although in absentia, suggest the presence of lateral genicular arteries, but none of them gives clear instructions about their localization for the safety of these vessels, and damage to these arteries is fraught with the above complications. Moreover, if we take into account the anatomical localization variability of these vessels, these recommendations fail to be safe regarding the blood supply to the patella.

Several authors have studied the safety of lateral genicular arteries during the lateral release in TKA. De Bell et al. tried to detect these arteries with the naked eye in a large cadaver study by simulating the lateral release of the patellar retinaculum. They were able to detect arteries in only three cases out of ten samples of the knee joint and all of them were SLGA [16].

Another cadaver study conducted by Lazaro et al. on the 21 knee joints showed that the medial parapatellar approach completely cuts off the medial genicular arteries and the main source of blood supply remains on the lateral side of the patella, along with the other small vessels of the anastomotic ring. In this study, the authors proposed to preserve/improve the blood supply to the patella by leaving intact more than 15 mm of soft tissue structures from the medial edge of the patella [4].

In another study devoted to the blood supply of the patella, much attention is paid to the sub-patellar zone, namely, the Hoffa’s fat pad, which also abundantly supplies blood to the patella. At the same time, they also indicate the need to preserve the superior lateral genicular artery, in terms of possible postoperative complications [28].

Our proposed method was successful in ten out of 12 cases by identifying at least one lateral genicular artery. The anatomical real-time localization of the lateral arteries that we found allows us to assume that if the lateral release protocol was followed blindly, all the vessels could be irreversibly damaged.

Our results confirm the data of some authors regarding the different locations and directions of the lateral genicular arteries, which makes it even more difficult to identify them without auxiliary tools (83.3%, transillumination method, versus 30%, with the naked eye) [16].

Imaging techniques could also be useful to prevent vascular damage during lateral release. Thus, preoperative contrasting magnetic resonance imaging would allow us to accurately localize the patellar vessels and dynamically monitoring their condition in the postoperative period. In comparison with this highly informative method, our method has several advantages: Firstly, the possibility of intraoperative accurate determination of the location and direction of the vessel after implantation of the components of the endoprosthesis. Secondly, the instrument set required to perform our method does not go beyond the arsenal of a modern operation room. Thirdly, the proposed method is performed without the use of contrast agents and is obviously free from dye-related complications [29, 30].

The limitations of our study were a small number of subjects (twelve knee joints). Therefore, our data at this stage may need to be interpreted with caution. We also limited the time to search for the lateral patellar arteries to five min and do not recommend wasting more time on visualization and identification of vessels since the elongation of the operation time correlates directly with a potentially challenging and catastrophic complication such as periprosthetic joint infection [31].

In two cases out of 12, we were unable to identify the lateral patellar arteries. Failure of detection in these cases could be because soft tissue structures did not allow sufficient visualization of vessels due to their thickening (hypertrophied joint capsule, thickening, and contracture of the lateral retinaculum). Possible relative factors that can affect visualization were the intensity of the light source, the illumination of the operating room for the most optimal visualization, and an increase in the temperature of the illuminated area. Further studies should be conducted to better understand and improve the utility of the proposed method. For an objective assessment of the blood supply to the lateral parapatellar area, the method of ultrasound dopplerography was used, since this method is the most accessible and sensitive regarding vessels.

## Conclusion

Lateral release of the patellar retinaculum should be carried out under careful monitoring and visualization of the lateral genicular arteries. Using the proposed transillumination method, an orthopaedic surgeon can perform a lateral release while preserving the lateral blood supply to the patella, when the medial side has already been sacrificed by the medial parapatellar approach. This method provides an opportunity to avoid iatrogenic devascularization of the patella, postoperative patellar complications, and, importantly, blood loss reduction in TKA.

## Data Availability

The data that supports the findings of this study are available on request from the corresponding author, S. M.

## References

[1] Kremers HM, Larson DR, Crowson CS (2015). Prevalence of total hip and knee replacement in the United States. J Bone Joint Surg Am.

[2] Singh JA (2011). Epidemiology of knee and hip arthroplasty: a systematic review. Open Orthop J.

[3] Ben-Shlomo Y, Blom A, Boulton C (2021). The National Joint Registry 18th Annual Report 2021.

[4] Lazaro LE, Cross MB, Lorich DG (2014). Vascular anatomy of the patella: implications for total knee arthroplasty surgical approaches. Knee.

[5] Assiotis A, To K, Morgan-Jones R (2019). Patellar complications following total knee arthroplasty: a review of the current literature. Eur J Orthop Surg Traumatol.

[6] Schiavone Panni A, Cerciello S, Del Regno C (2014). Patellar resurfacing complications in total knee arthroplasty. Int Orthop.

[7] Maheshwari AV, Tsailas PG, Ranawat AS, Ranawat CS (2009). How to address the patella in revision total knee arthroplasty. Knee.

[8] Postler A, Lützner C, Beyer F et al (2018) Analysis of total knee arthroplasty revision causes. BMC Musculoskelet Disord 19. 10.1186/S12891-018-1977-Y 10.1186/s12891-018-1977-y PMC5813428 29444666

[9] Schroer WC, Berend KR, Lombardi AV (2013). Why are total knees failing today? Etiology of total knee revision in 2010 and 2011. J Arthroplasty.

[10] Yoo JD, Kim NK (2015). Periprosthetic fractures following total knee arthroplasty. Knee Surg Relat Res.

[11] Lachiewicz PF, Soileau ES (2006). Patella maltracking in posterior-stabilized total knee arthroplasty. Clin Orthop Relat Res.

[12] Motsis EK, Paschos N, Pakos EE, Georgoulis AD (2009). Review article: patellar instability after total knee arthroplasty. J Orthop Surg (Hong Kong).

[13] Yang CC, McFadden LA, Dennis DA (2008). Lateral retinacular release rates in mobile- versus fixed-bearing TKA. Clin Orthop Relat Res.

[14] Kusuma SK, Puri N, Lotke PA (2009). Lateral retinacular release during primary total knee arthroplasty: effect on outcomes and complications. J Arthroplasty.

[15] da Fonseca LPRM, Kawatake EH, de C Pochini A (2017). Lateral patellar retinacular release: changes over the last ten years. Rev Bras Ortop (English Ed).

[16] DeBell H, Pinter Z, Pinto M (2019). Vascular supply at risk during lateral release of the patella during total knee arthroplasty: a cadaveric study. J Clin Orthop Trauma.

[17] Kohn MD, Sassoon AA, Fernando ND (2016). Classifications in brief: kellgren-lawrence classification of osteoarthritis. Clin Orthop Relat Res.

[18] Cho WS, Woo JH, Park HY (2011). Should the “no thumb technique” be the golden standard for evaluating patellar tracking in total knee arthroplasty?. Knee.

[19] Naranje S, Lendway L, Mehle S, Gioe TJ (2015). Does Operative time affect infection rate in primary total knee arthroplasty?. Clin Orthop Relat Res.

[20] Peersman G, Laskin R, Davis J (2006). Prolonged operative time correlates with increased infection rate after total knee arthroplasty. HSS J.

[21] Wheeless CR, Nunley JA, Urbaniak JR, of Orthopaedic Surgery DUMCD (2016). Wheeless’ Textbook of Orthopaedics.

[22] Masoni V, Giustra F, Bosco F et al (2023) Periprosthetic patella fractures in total knee replacement and revision surgeries: how to diagnose and treat this rare but potentially devastating complication—a review of the current literature. Eur J Orthop Surg Traumatol. 10.1007/s00590-023-03535-9 10.1007/s00590-023-03535-9 PMC10504128 37000239

[23] Deans J, Scuderi GR (2021). Classification and management of periprosthetic patella fractures. Orthop Clin North Am.

[24] Narkbunnam R, Electricwala AJ, Huddleston JI (2019). Suboptimal patellofemoral alignment is associated with poor clinical outcome scores after primary total knee arthroplasty. Arch Orthop Trauma Surg.

[25] Strachan RK, Merican AM, Devadasan B (2009). A technique of staged lateral release to correct patellar tracking in total knee arthroplasty. J Arthroplasty.

[26] Maniar RN, Singhi T, Rathi SS (2012). Surgical technique: lateral retinaculum release in knee arthroplasty using a stepwise, outside-in technique knee. Clin Orthop Relat Res.

[27] Maniar AR, Maniar RN (2021). A simple, stepwise, outside-in technique for lateral retinacular release for management of patellar maltracking during total knee arthroplasty. J Am Acad Orthop Surg.

[28] Nemschak G, Pretterklieber ML (2012). The patellar arterial supply via the infrapatellar fat pad (of Hoffa): a combined anatomical and angiographical analysis. Anat Res Int.

[29] Rozenfeld MN, Podberesky DJ (2018). Gadolinium-based contrast agents in children. Pediatr Radiol.

[30] Penfield JG (2008). Nephrogenic systemic fibrosis and the use of gadolinium-based contrast agents. Pediatr Nephrol.

[31] Ravi B, Jenkinson R, O’Heireamhoin S (2019). Surgical duration is associated with an increased risk of periprosthetic infection following total knee arthroplasty: a population-based retrospective cohort study. EClinicalMedicine.

